# γδ T Cell-Mediated Immunity to Cytomegalovirus Infection

**DOI:** 10.3389/fimmu.2017.00105

**Published:** 2017-02-09

**Authors:** Camille Khairallah, Julie Déchanet-Merville, Myriam Capone

**Affiliations:** ^1^Immunoconcept, CNRS UMR 5164, Bordeaux University, Bordeaux, France

**Keywords:** γδ T cells, cytomegalovirus, bone marrow and organ transplantation, antiviral immunity, memory T cells

## Abstract

γδ T lymphocytes are unconventional immune cells, which have both innate- and adaptive-like features allowing them to respond to a wide spectrum of pathogens. For many years, we and others have reported on the role of these cells in the immune response to human cytomegalovirus in transplant patients, pregnant women, neonates, immunodeficient children, and healthy people. Indeed, and as described for CD8^+^ T cells, CMV infection leaves a specific imprint on the γδ T cell compartment: (i) driving a long-lasting expansion of oligoclonal γδ T cells in the blood of seropositive individuals, (ii) inducing their differentiation into effector/memory cells expressing a T_EMRA_ phenotype, and (iii) enhancing their antiviral effector functions (i.e., cytotoxicity and IFNγ production). Recently, two studies using murine CMV (MCMV) have corroborated and extended these observations. In particular, they have illustrated the ability of adoptively transferred MCMV-induced γδ T cells to protect immune-deficient mice against virus-induced death. *In vivo*, expansion of γδ T cells is associated with the clearance of CMV infection as well as with reduced cancer occurrence or leukemia relapse risk in kidney transplant patients and allogeneic stem cell recipients, respectively. Taken together, all these studies show that γδ T cells are important immune effectors against CMV and cancer, which are life-threatening diseases affecting transplant recipients. The ability of CMV-induced γδ T cells to act independently of other immune cells opens the door to the development of novel cellular immunotherapies that could be particularly beneficial for immunocompromised transplant recipients.

## Introduction

Cytomegaloviruses (CMVs) belong to the betaherpesvirus family and infect different species including rodents, non-human primates, and humans. The human cytomegalovirus (HCMV), also known as human herpesvirus 5 (HHV5), is an extremely widespread pathogen that infects from 30 to 90% of individuals. CMVs are highly species specific, having coevolved and adapted to their respective host. Thus, HCMV is unable to establish a productive infection in mice. Yet, human and murine CMV (MCMV) share many biological properties: (i) they present comparable structures and some viral proteins are homologous between human and mouse (1, 2); (ii) they show similar tissue tropism (3, 4); (iii) they induce similar pathologies in immunocompromised hosts (e.g., pneumonitis or hepatitis) (5–9), justifying MCMV infection of mice a widely used *in vivo* model to study CMV pathogenesis and antiviral immunity.

Cytomegaloviruses are naturally transmitted through direct contact with body fluids such as saliva, urine, sperm, and breast milk. In immunocompetent hosts, CMV infection is usually asymptomatic, but some individuals may experience mild symptoms (10). However, the resolution of primary CMV infection does not result in complete elimination of the virus. Instead, CMV persists within its host in a latent form in hematopoietic and, likely, endothelial cells (11). Reactivation of viral gene expression occurs sporadically and might be initiated by chromatin remodeling (12) [for review on latency, see Ref. (13–15)]. The mechanism controlling the exit from CMV latency depends on both the differentiation status of the latently infected cells, and on the immune status of the host. Keeping CMV asymptomatic thus requires a robust and well-orchestrated immune response.

The immunosuppressive or hematoablative therapy applied in solid organ transplantation (SOT) or hematopoietic stem cell transplantation (HSCT) render patients susceptible to opportunistic pathogens, with CMV infection being the most common. CMV can cause either a viral syndrome (with fever, leukopenia) or a tissue-invasive disease (such as hepatitis, pneumonitis). Fortunately, the clinical effects of CMV infection have been greatly reduced by preemptive, prophylactic, and curative therapies, such as the development of CMV viremia detection (antigenemia and PCR) and of anti-CMV antivirals (ganciclovir, valganciclovir) (16). Nonetheless, CMV continues to be one of the leading causes of morbidity, due to the toxicities of antiviral drugs, to the emergence of antiviral resistance (17–19), to the “indirect effects” of CMV infection (20), and opportunistic infections (21, 22). Consequently, there is growing interest in evaluating cell-mediated immunity to improve the diagnosis and management of CMV infection.

**Figure 1:**
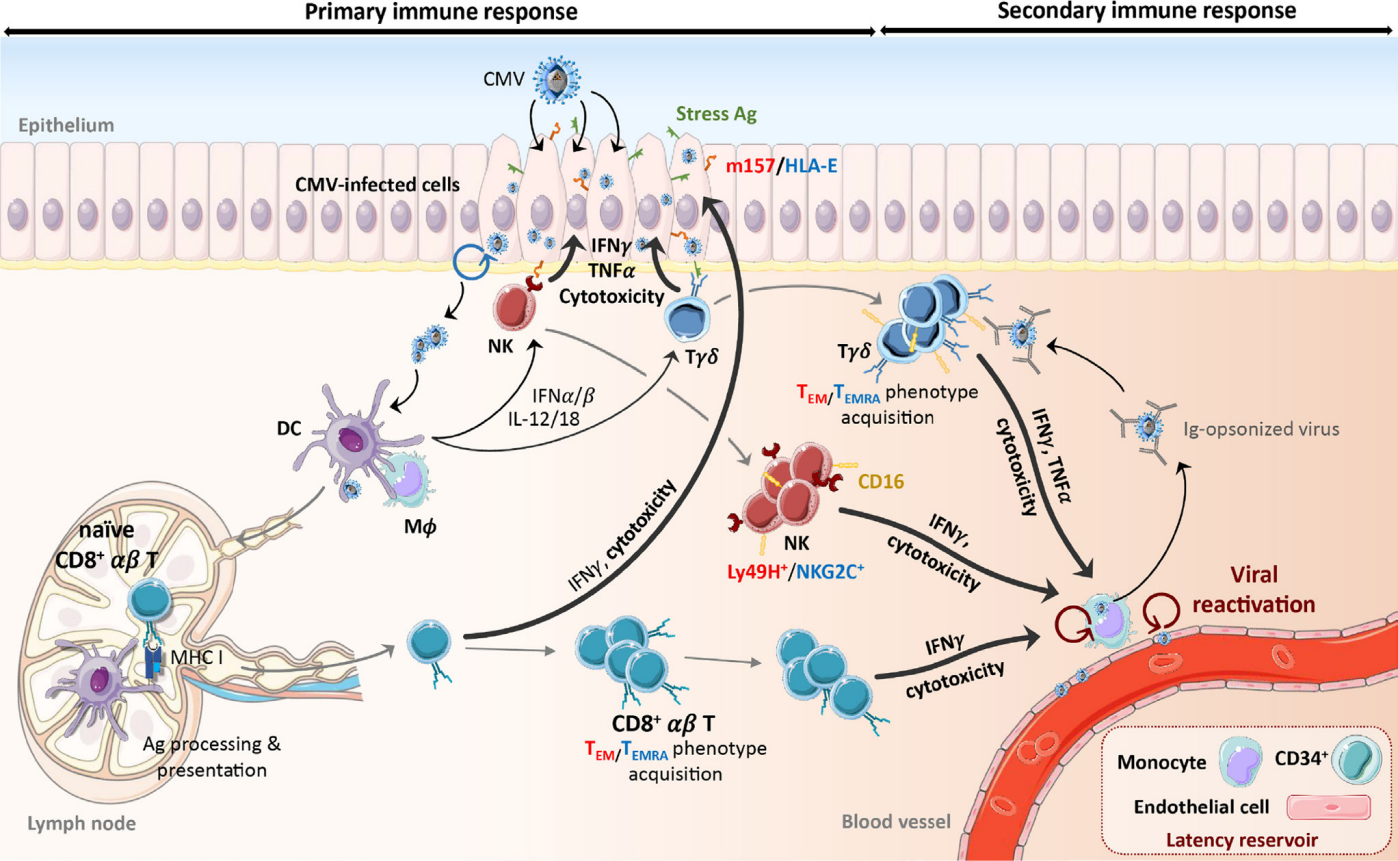
**Schematic representation of the primary and secondary response to CMV**. Early during primary CMV infection, phagocytes and DCs are activated through TLRs and nucleic acid sensors by viral products and secrete pro-inflammatory cytokines (IFNαβ, IL-12, and IL-18) that induce NK cell and γδ T cell activation. Recognition of the protein m157 (C57BL/6 mouse) and HLA-E (human) or stress-induced ligands expressed by infected cells also stimulates NK cells and γδ T cells, respectively. This leads to the expansion of Ly49H^+^ (mouse) or NKG2C^+^ (human) NK cells and T_EM_ (mouse) or CD16^+^ T_EMRA_ (human) γδ T cells that persist over the long term. Activation of DCs leads to their maturation and migration to lymph nodes. Cross-presentation of viral peptides to naïve CD8^+^ αβ T cells induces their differentiation into T_EM_ or T_EMRA_, expansion and acquisition of effector functions. Activated NK cells and αβ and γδ T cells can lyse and eliminate CMV-infected cells or control viral replication through secretion of anti-viral cytokines (e.g., IFNγ, TNFα). Despite the establishment of this immune response, CMV persists in its host. During viral reactivation episodes, CMV-induced immune cells react quickly to the presence of virions through the recognition of m157/HLA-E, stress antigens, or viral peptides. In addition, IFNγ secretion by CMV-elicited γδ T cells can be induced by CD16 interaction with Ig-opsonized viruses. The following color code has been used to distinguish mouse and human molecules or phenotypes: red color-mouse, blue color-human. Ag, antigen; CMV, cytomegalovirus; DC, dendritic cell; IFN, interferon, Ig, immunoglobulin; IL, interleukin; Mϕ, macrophage; NK, natural killer cell; T_EM_, effector memory T cell; T_EMRA_, CD45RA^+^ effector memory T cell; TLR, toll-like receptor.

**Figure 2:**
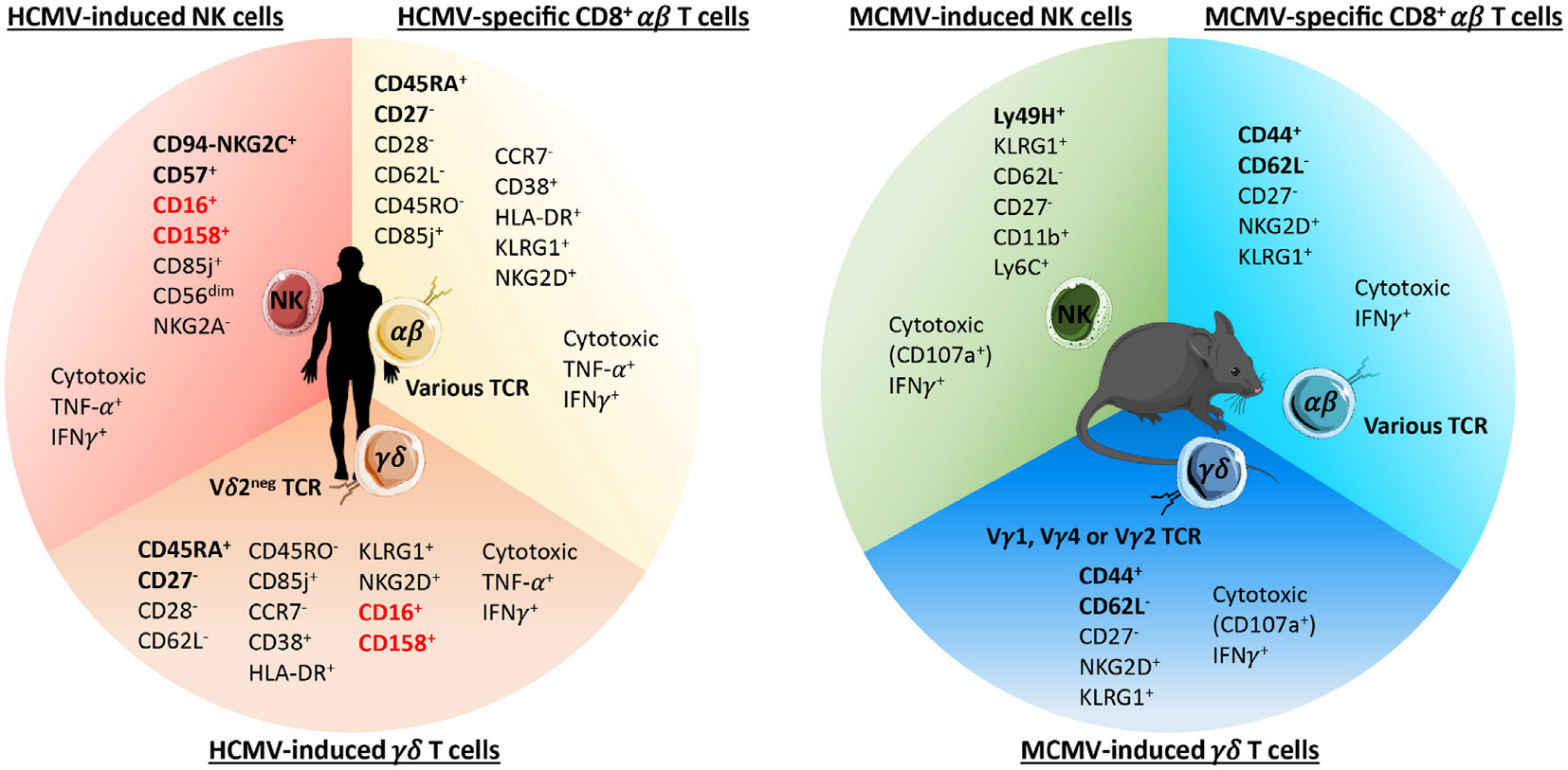
**Phenotypes of long-term cytomegalovirus (CMV)-induced NK, CD8^+^ αβ, and γδ T cells in humans and C57BL/6 mice**. The main phenotypic and functional features of human (left panel) and mouse (right panel) CMV-induced NK and γδ T cells and CMV-specific CD8^+^ αβ T cells are listed. The surface markers commonly used to identify each population are emphasized in bold. As depicted, human and murine CMV-induced γδ T cells express an effector/memory phenotype closely related to CMV-specific CD8^+^ αβ T cells. In addition, human CMV-induced Vδ2^−^ γδ T cells also shared some features (highlighted in red) with CMV-induced NK cells among which the expression of CD16 and CD158.

Cell-mediated immunity to CMV is among the most robust ever documented. Before focusing on γδ T cells, we will provide a quick overview of the NK and CD8^+^ αβ T cell responses to CMV in humans and mice. These responses are depicted in Figures 1 and 2.

## Cellular Immunity During Acute CMV Infection in Immune-Competent Mice

The mouse model of CMV infection has been useful to study the kinetics of immune effectors responses in organs, particularly in the liver, spleen, and lungs, which are important targets of CMV. Early post MCMV entry, phagocytes and dendritic cells (DCs) are activated through the recognition of viral products by toll-like receptors (TLR) (23, 24) and the interferon-inducible protein AIM2, which binds double-stranded DNA (25). This leads to the release of type I interferons (IFNs) and inflammatory cytokines, among which are interleukin (IL)-12 and IL-18 (26–28). These mediators induce early IFNγ production and cytolysis by NK cells (29, 30) (Figure 1). Infection of mice with MCMV has provided direct evidence of the importance of this subset in CMV clearance and protection. In contrast to BALB/c mice, C57BL/6 mice are highly resistant to CMV, due to expression of Ly49H on 50% of NK cells, an activating receptor that recognizes the virally encoded m157 viral protein on the surface of infected cells (31–34). Over the first week of MCMV infection, Ly49H^+^ NK cells expand significantly in the liver and spleen, and begin expressing the inhibitory receptor KLRG1 (35–37). Establishment of primary CMV infection also drives DCs maturation. Presentation and/or cross-presentation of viral peptides to CD4^+^ and CD8^+^ αβ T cells induces their differentiation and effector function. According to a report by Schlub et al., the kinetics of NK and T cell proliferation during acute MCMV infection are concomitant and peak at day 7; however, NK cell contraction after the peak is slower than that of T cells (38).

While the role of NK cells in early control of CMV was clearly evidenced in mice, CD3ϵ^−/−^ mice succumbed to MCMV infection about 4 weeks after exposure. These results emphasize the importance of T cells in long-term control of MCMV. Studies in MCMV-infected, T-cell-deficient mice also revealed redundancy between T cell effectors [CD4^+^ versus CD8^+^ (39, 40), αβ versus γδ (41)], likely because they share important features for host protection.

## Cellular Immunity During Acute HCMV Infection

The immune response against human CMV is, in its main steps, similar to the one observed in the mouse and is largely based on the triptych “Dendritic cells (DC)—NK cells—αβ T cells” (42, 43). HCMV entry occurs in concert with immune detection through TLR (44, 45) and nucleic acid sensors. The gamma-interferon inducible protein IFI16 was shown to play a crucial role as a viral DNA sensor in the first hours postinfection (46), but also acts as a repressor of viral gene transcription in the later stages (47). Recognition of viral products by TLR and DNA sensors induces the production of inflammatory cytokines and type I IFN by innate effectors, and subsequent activation of NK and αβ T cells. However, in healthy human subjects, the onset of primary HCMV infection typically goes unnoticed making it difficult to analyze the kinetics of immune effectors. The majority of studies regarding the early phases of the immune response to the virus have been carried out in HCMV-naive recipients (R^−^) of organ transplant from CMV-seropositive donors (D^+^). The situation is different from that of healthy individuals since transplant recipients are subjected to: (i) immunosuppressive drugs that cause lymphopenia followed by homeostatic proliferation of lymphocytes, and (ii) antiviral therapies that influence the virus/lymphocytes ratio and subsequent activation of lymphocytes. One to two weeks after detection of HCMV viremia in blood, a NKG2C^+^ NK cell population preferentially expands and upregulates NKG2C and CD57 (48). NKG2C^+^ NK cells have been considered the human counterparts of murine Ly49H^+^ NK cells because of their reactivity against HCMV-infected cells and their memory function. However, in contrast to Ly49H, NKG2C recognize the self-ligand HLA-E (49) (Figures 1 and 2). HCMV-specific αβ T lymphocytes also appear in blood after the peak of CMV replication, with variable kinetics dependent on the patient and the immunosuppressive environment. The HCMV-specific αβ T cell responses that dominate during the acute phase are typified by classical expansion, contraction, and formation of long-term effector and central memory pools (50–52) [reviewed in Ref. (53)].

## Latent CMV Infection and Long-Term Antiviral CD8^+^ T Cell Response

During latent CMV infection in both humans and mice, a progressive and prolonged expansion of CMV-specific CD8^+^ αβ T cells has been observed, a phenomenon called “memory inflation” [reviewed in Ref. (54–56)]. Only a few epitopes drive memory inflation, derived from both early and late CMV gene products. Memory inflation was primary described in BALB/c mice by Holtappels et al., who showed an enrichment of CD62L^−^ CD8^+^ T cells specific to IE1 (m123/pp89), during latent MCMV infection in the lungs (57). In C57BL/6 mice, four distinct patterns were discerned, based on the epitope-specific CD8^+^ T cell responses during acute and persistent MCMV infection (58): (i) the responses to M45 and M57 displayed the classic kinetics of expansion, contraction, and stable memory, (ii) the response to m139 peaked at day 7, rapidly contracted, then underwent memory inflation, (iii) the response to M38 peaked at day 14 but underwent only limited contraction before reaching a long-term plateau, (iv) the responses to IE3 epitopes were above background until day 35, but became robust ≥4 months after infection. In subsequent studies, the patterns exemplified by M45 and M38 were considered to be non-inflationary and inflationary, respectively. Interestingly, the C57BL/6 CD8^+^ T cell response to m139 is reminiscent to the one described for IE1/pp89 and m164 in BALB/c mice (59). Human inflationary CD8^+^ T cells recognize both IE1- and pp65-specific epitopes. In infants who mounted acute CD8 T cell responses, it was found that the IE1-specific response was always larger than the pp65-specific response by 1 year of age, regardless of which Ag was immunodominant upon initial infection (60). pp65-specific inflationary CD8^+^ T cells display an oligoclonal but diverse αβ T cell receptor (TCR) repertoire that can be renewed upon antigen (Ag) reexposure (61).

In humans, inflationary CD8^+^ T cells use the longer CD45 isoform (CD45RA), reminiscent of terminally differentiated cells. However, they show no evidence of T cell exhaustion and remain functional. Human and murine CMV-specific T cells exhibit several other features associated with T cell maturation (62, 63). These include downregulated expression of the coreceptors CD27 and CD28, and the expression of effector molecules such as perforin and granzyme (Figure 2). In C57BL/6 mice, M38-specific CD8^+^ T cells express an effector memory (EM) phenotype (CD62L^−^CCR7^−^CD27^−^), while chronic CD8^+^ T cells specific for M45 regained CD62L expression, typical of TCM (58). In comparison to long-lived CD8^+^ TCM, inflationary CD8^+^ T cells display higher expression of inhibitory receptors such as KLRG1 (63, 64) (Figure 2). According to mouse studies, the drivers of memory CD8^+^ T cell inflation are latently infected non-hematopoietic cells (65, 66). CD8^+^ T cells that dominate the chronic phase of MCMV infection are short lived and continuously turned over (54, 67, 68). The likely source of inflationary CD8^+^ T cells is CD27^+^KLRG1^−^ cells, because of their high proliferative and self-renewal potential (69).

## Latent CMV Infection and Long-Term Antiviral NK Cell Response

Contrasting initial studies suggesting a short life span for NK cells, a set of recent studies describe long-term maintenance of memory-like NK cells in MCMV- and HCMV-infected hosts (70, 71). After adoptive transfer in DAP12- and Ly49H-deficient mice, Ly49H^+^ cells undergo a robust clonal expansion followed by contraction and persistence for 70 days (72). This memory population has self-renewing capacity and is 10 times more potent in conferring protection against reinfection when compared to naïve cells. In mice, memory NK cells express high levels of KLRG1, low levels of CD27 (Figures 1 and 2), and are derived from KLRG1-negative progenitors with high proliferative potential (73). In humans, NKG2C^+^CD57^+^ NK cells express CD85j and can represent up to 70% of the total population of NK cells in HCMV-seropositive individuals. Their memory potential was suggested in HCMV-seropositive stem cell recipients who received a HCMV-seropositive (D^+^R^+^) or seronegative graft (D^−^R^+^). Importantly, NKG2C^+^ NK cells transplanted from D^+^ exhibit heightened function in response to a secondary CMV event compared with NKG2C^+^ NK cells from D^−^. Memory NKG2C^+^CD57^+^ NK cells display a mature phenotype, they are CD56^dim^, lack NKG2A, and express CD158b (48, 74, 75) (Figure 2).

More generally, during HSCT, the incidence of virus recurrence and disease is highest in the combination of an HCMV-negative donor (D^−^) and an HCMV-positive recipient (R^+^) (D^−^R^+^ > D^+^R^+^ > D^+^R^−^), while just the opposite is true in the case of SOT (D^+^R^−^ > D^+^R^+^ > D^−^R^+^). These risk assessments support the suggestions that (i) HCMV-reactivation occurs in latently infected tissues even in the case of HSCT and (ii) the development of antiviral immune memory responses (of donors in case of HSCT, or recipients in case of SOT) is a good prognostic factor against CMV disease.

## CD8^+^ T Cell Immunotherapy of CMV Disease in HSCT

The work by the Reddehase group in the mouse model of HSCT contributed substantially to provide a proof of concept for CD8^+^ T-cell-based immunotherapy [for review, see Ref. (76–78)]. BALB/c mice received hematoablative, total-body γ irradiation followed by syngenic HSCT the same day as virus inoculation. This reproduced the timeframe of early-onset CMV disease in HSCT patients (1–4 months). In this model, infusion of MCMV-specific CD8^+^ T cells accelerated the resolution of primary infection and limited the establishment of viral latency (79). Moreover, MCMV was shown to infect bone marrow (BM) stromal cells and to interfere with T cell reconstitution after HSCT. CD8^+^ T cell immunotherapy thus tilts the balance in favor of viral control and gives a window for immune reconstitution. In the absence of adoptive T cells therapy, mortality can be prevented by transferring high doses of HSC into the host. By means of a recombinant virus in which four (BALB/c) immunodominant epitopes (IDEs) were functionally deleted, the Reddehase group elegantly showed that reconstitution of IDE-specific CD8^+^ T cells is not essential for antiviral control in infected HSCT hosts (80). Efficient protection in the absence of IDE was also evidenced in the CD8^+^ T cells adoptive transfer scenario (81).

Developing novel anti-HCMV therapies constitutes a major issue in transplantation. Adoptive transfer of HCMV-specific T cells from donors was shown to reduce the risk for HCMV disease in HSCT (82–86) and, more recently, SOT (87, 88). Multiple parameters that determine the efficacy of adoptive CD8^+^ T cells antiviral therapy are still under consideration, among which are (i) their antiviral function, (ii) their migratory capacity, (iii) their memory and self-renewing potential (77, 89), and (iv) their TCR avidity by monitoring dissociation (*k*_off-_rate) of truly monomeric peptide–MHC complexes bound to surface-expressed TCRs (90).

Allogeneic HSCT can be used to treat otherwise incurable leukemia. Consequently, novel strategies in HSCT aim at reducing graft-versus-host disease, while maintaining immunological anti-leukemia and anti-infectious activity. Recent investigations aim at evaluating the potential use of NK cells (91, 92) and innate like effectors (93, 94) in this context.

## γδ T Cells, Antigen Recognition, and Effector Fate

γδ T lymphocytes contribute to both anti-infectious and antitumor immune responses and display unique properties rendering them attractive targets for immunotherapy (95–98). Although they share important functions with αβ T cells, γδ T cells are distinct from αβ T cells most notably in antigen recognition and effector fate development. In contrast to αβ T cells, γδ T cells are not restricted by the major histocompatibility complex (MHC). The nature of ligands recognized by the γδ TCR is quite diverse including MHC-related and unrelated proteins, as well as low molecular weight non-peptidic ligands often found associated to presenting molecules [reviewed in Ref. (99–101)].

γδ T cells are the first T cells to appear in the fetal thymus. As the differentiation of αβ T cells progresses, the relative proportion of γδ T cells decreases. In adult human peripheral blood, γδ T cells comprise approximately 4% of total CD3^+^ cells. γδ T cells home to similar peripheral sites as αβ T cells, in both lymphoid organs and tissues. They are generally found in lower proportions than αβ T cells, with the exception of epithelial sites where mouse γδ T cell subtypes home specifically during ontogeny and can reach 40% (intestine) and 100% (epidermis) of T lymphocytes. Most of γδ T cells found in organs from naïve mice display a functional polarization that is acquired during thymic selection. Expression of the costimulatory receptor CD27 segregates IL-17-producing (CD27^−^) and IFNγ-producing (CD27^+^) γδ T cells (102). CD27^−^ γδ T cells are nonetheless endowed with functional plasticity and may produce IFNγ under local inflammatory conditions (103). In the periphery, functional orientation of γδ T cells depends on the microorganism encountered, with IFNγ- and IL-17-production dominating antiviral and antibacterial responses, respectively (99).

γδ T lymphocytes are subdivided into subsets according to the nature of their TCR and cytokine production preferences [for review, see Ref. (99, 104–106)]. In mice, the fetal thymus gives rise consecutively to Vγ5^+^ (Vδ1^+^) and Vγ6^+^ (Vδ1^+^) γδ T cells, that home to the skin (Vγ5^+^ dendritic epidermal T cells or DETC), lungs, and uterus (Vγ6^+^), respectively. These cells are pre-committed to IFNγ (CD27^+^ Vγ5^+^) and IL-17 (CD27^−^ Vγ6^+^) production. Before birth develop IFNγ-producing CD27^+^ NK1.1^+^ Vγ1^+^ (Vδ6.3^+^/6.4^+^) cells, as well CD27^−^ IL17-producing Vγ4^+^ (Vδ5^+^) cells (107). The (semi)-invariant nature of their TCR and response pattern contribute to the classification of these fetus-derived γδ T cells as “innate-like.” They show rapid responsiveness to innate stimuli such as upregulation of the expression of NKG2D ligand for DETC (108), IL-1β plus IL-23 for CD27^−^ Vγ6^+^ cells (109), IL-18 plus IL-12 for CD27^+^ NK1.1^+^ Vγ1^+^, and CD27^+^ CD45RB^high^ Vγ4^+^ cells (110, 111). Innate-like γδ T cells are activated in the thymus and display an EM CD44^+^CD62L^−^ phenotype, unlike “naïve” polyclonal CD44^−^CD62L^+^ Vγ1^+^ and Vγ4^+^ γδ T lymphocytes generated during adulthood. Naïve γδ T cells are mostly found in peripheral organs and blood. They are activated in the periphery after Ag exposure and display a functional plasticity. Other tissue-specific γδ T cell populations, including intraepithelial intestinal γδ T cells develop throughout adulthood. Intraepithelial intestinal γδ T cells express TCRs mainly composed of Vγ7.

In human, γδ T cells are divided in two subsets, the Vγ9^+^Vδ2^+^ T cells that are found predominantly in the blood, and all the other γδ T cells (collectively called Vδ2^−^ γδ T cells, and mainly composed of Vδ1^+^ and Vδ3^+^ T cells) that are primarily located in tissues, particularly in epithelia. Only Vγ9^+^Vδ2^+^ T cells are activated through the TCR, and this is by small phosphorylated metabolites from the isoprenoid biosynthesis pathway (called phosphoantigens). Antigens recognized by Vδ2^−^ γδ TCRs are largely unknown; however, a small subset of Vδ1 TCRs has been shown to recognize CD1d, both associated with lipids or not (112).

## Response of γδ T Cells to HCMV

The first evidence showing the mobilization of γδ T cells against HCMV was obtained in kidney transplant patients in our laboratory in 1999 (113). In those immunosuppressed patients, HCMV infection leads to a strong increase (in proportion and number) of γδ T cells in the blood circulation, which persisted long term (114–116). Surprisingly, this expansion does not include the major γδ T cell subset present in the blood, namely, the Vγ9^+^Vδ2^+^ T lymphocytes. Indeed, HCMV-induced γδ T cells have been shown to express mainly the Vδ1 or the Vδ3 chain, and in some cases the Vδ5 chain. This rise of circulating γδ T cells correlated with the resolution of the infection, supporting their antiviral role (117).

Since these first reports, the expansion in blood and antiviral function of Vδ2^−^ γδ T cells during HCMV infection has been shown in several other contexts of immunosuppression linked to organ and BM transplantation (118–120), in pregnant women (121, 122), and in children with a severe combined immunodeficiency (123, 124). Interestingly, this expansion is also observed in seropositive, healthy individuals illustrating that γδ T cells are not only mobilized in people with a defective immune response (119, 125). In otherwise healthy adults, HCMV was shown to prevent the decline of Vδ2^−^ γδ T cells in the blood normally observed in the elderly (122, 126). Interestingly, Vermijlen’s team reported that, in addition to their role in adults, γδ T cells can participate in antiviral response early in life. In their report, they observed a γδ T cell response by the fetus during *in utero* HCMV infection (127). This response is qualitatively different from that observed in adults, since it involves Vδ2^+^ cells in addition to Vδ1^+^ and Vδ3^+^ cells. Fetal HCMV-specific γδ T lymphocytes are Vγ9^−^ cells and express a public Vγ8^+^Vδ1^+^ TCR that has never been found in adults during HCMV infection (128). These differences show the capacity of HCMV to mobilize different repertoires of γδ T cells at different periods of life, highlighting the close relationship between this virus and γδ T cells.

## Evidencing the Protective Anti-CMV Role of γδ T Cells in Mice

Murine and human γδ T cells are closely related in many aspects including their predominant tissue localization (129), their ability to recognize non-MHC restricted viral antigens (130, 131), and their participation to the immune response against certain herpesviruses (132, 133). Infection of mice with MCMV thus appears as an interesting model to help decipher the role of γδ T cells in the immune response to CMV and extend the observations made in humans.

Using partially immunodeficient mice in the C57BL/6 background, both our research team and Mach and Winkler have recently proven the protective antiviral function of γδ T cells during MCMV infection. Despite the reported importance of conventional αβ T cells in the control of MCMV (8, 78, 134), we showed that murine γδ T lymphocytes are capable of protecting αβ T cell-deficient mice (i.e., TCRα^−/−^ mice) against MCMV-induced organ damage and death (41). In contrast, CD3ϵ^−/−^ mice (that lack both αβ and γδ T cells) died around 1 month postinfection and show liver and lungs pathology, highlighting the absolute requirement of a T cell response in antiviral protection. The protective role of γδ T cells neither rely on B lymphocytes nor Ly49H^+^ NK cells, as CD4-depleted CD8^−/−^ JHT mice (deficient for both CD8^+^ αβ T and B cells) survived upon challenge with a MCMV strain lacking the NK cell-activating m157 viral protein (135). In both TCRα^−/−^ and CD4-depleted CD8^−/−^ JHT mice, viral loads decreased about 2 weeks postinfection, concomitantly with γδ T cell expansion in various CMV target organs including spleen, liver, and lungs (41, 135). The rise in γδ T cell number is at least partially due to the local proliferation of γδ T cells, as a substantial fraction of these cells incorporated BrdU after 14 days of infection (135). γδ T cell expansion was also evidenced in immunocompetent MCMV-infected hosts (136, 137), even though TCRδ^−/−^ mice survived MCMV infection (41). The protective capacity of MCMV-induced γδ T cells isolated from both αβ-deficient and immunocompetent mice was confirmed by transfer into Rag^−/−^ and Rag^−/−^γc^−/−^ highly immunodeficient hosts that survived MCMV infection (41, 135). These results extend human studies and show that γδ T cells are an integral part of the immune response against CMV.

Although dispensable in immunocompetent hosts, γδ T cells could become essential in particular contexts of immunodeficiency. Highlighting this assessment, immunodeficient children carrying a hypomorphic recombination activating gene (RAG)-1 or a TCRα subunit constant gene (TRAC) mutation are relatively well protected against HCMV in spite of their deficiency in αβ T lymphocytes (123, 124, 138).

## Similarities Between γδ and αβ T Cells in the Response to HCMV

Human cytomegalovirus-induced Vδ2^−^ γδ T lymphocytes and antiviral αβ T cells share common features evocative of an adaptive-like immune response. First, the rise of circulating γδ T cells is strictly correlated to HCMV infection, as conversely low γδ T cell percentages are correlated with other viral infections [herpes simplex virus (HSV), Epstein–Barr virus, influenza, and varicella-zoster] (114). Second, γδ and CD8^+^ αβ T cells follow similar expansion kinetics in infected kidney transplant patients and pregnant women (121, 122, 139). Third, HCMV infection shapes the γδ TCR repertoire toward oligoclonality, even monoclonality in some extreme cases, while no repertoire restriction is observed in HSV-infected compared to seronegative individuals (125). Public HCMV-specific αβ TCRs have been described in adults infected with the virus (140). Although this has not been reported in adults for γδ TCRs, it is noteworthy that neonatal infection induces an enrichment of a public Vγ8Vδ1-TCR found in all the infected neonates (127). Thus, it is conceivable that HCMV-expanded γδ T cells are selected in an antigen-dependent manner, as described for HCMV-specific CD8^+^ αβ T cells. Consistent with this, recognition of HCMV-infected cells by γδ T cells isolated from HCMV-infected individuals involves the γδ TCR (114, 120, 141, 142).

Finally, HCMV-induced γδ T cells express an effector/memory T_EMRA_ phenotype, defined as CD45RA^+^CD27^−^CD28^−^CD62L^−^CD45RO^−^CCR7^−^CD38^+^HLA-DR^+^ (114, 122, 125, 139) and strictly similar to the one described for HCMV-specific CD8^+^ αβ T cells (143, 144) (Figure 2). αβ and γδ T cells induced upon HCMV infection also share expression of regulatory receptors such as KLRG1 (64, 127) (Figure 2), probably involved in the control of their expansion. The memory potential of T_EMRA_ Vδ2^−^ γδ T cells is suggested by a faster recall response of these cells and better infection resolution in transplant patients experiencing a secondary (D^+^R^+^) versus primary (D^+^R^−^) CMV infection (125). Interestingly, long-term expansion of T_EMRA_ Vδ2^−^ γδ T cells evokes the inflationary phenomenon observed for HCMV-specific CD8^+^ αβ T cells. An accentuation of HCMV-induced T_EMRA_ γδ T cell proportion has been reported in elderly (122, 145) as previously shown for HCMV-specific αβ T cells [reviewed in Ref. (146–148)].

## Mouse and Human CMV-Induced γδ T Cells Share Adaptive-Like Features

γδ T cells induced during MCMV infection share many characteristics with HCMV-expanded Vδ2^−^ γδ T cells. First, several subsets are involved in the response to CMV in both species {Vγ1, Vγ2, and Vγ4 in mice [nomenclature described in Ref. (149)] and Vδ1, Vδ3, and Vδ5 in humans} (41, 135, 136). Second, the expansion kinetics of γδ T cells in MCMV-infected mice and in HCMV-infected patients was similar to the one reported for conventional αβ T cells (58, 117, 150). Third, MCMV-induced Vγ1^+^ and Vγ4^+^ T cells acquired an EM phenotype that remained stable over time (41), as observed for MCMV-specific CD8^+^ αβ T cells (151), and reminiscent to HCMV-specific γδ and αβ T cells (Figure 2).

We hypothesize that a non-negligible part of MCMV-induced EM γδ T cells comes from naïve adaptive-like γδ T cells, mostly composed of Vγ1^+^ and Vγ4^+^ subsets and generated in the thymus after birth. As opposed to innate-like γδ T cells, adaptive-like γδ T cells display a more diverse TCR repertoire and have a delayed response due to their need for TCR-dependent priming to acquire their effector function (152, 153). The implication of these subsets in antiviral protection is further suggested by survival of BM transplant CD3ϵ^−/−^ recipients that received γδ T cell precursors from TCRα^−/−^ C57BL/6 mice (41). In this scenario, CMV infection occurs after immune reconstitution. In contrast, in the BALB/c mouse model described earlier, CMV infection is concomitant with HSCT in order to mirror early CMV reactivation posttransplantation. In these settings, depletion of CD8^+^ T cells during immune reconstitution (days 7 and 14 postinfection) was lethal [reviewed in Ref. (78)]. Reconstitution of sufficient numbers of protective γδ T cells (and of other immune subsets) might take too long to counteract the spread of the virus. It would be interesting to test whether this holds true regardless of the mouse genetic background, since C57BL/6 and BALB/c mice display strain-specific immunity to CMV. Dispensability of T/NK cells was evidenced in adoptive transfer experiments using MCMV-primed γδ T cells. Thus, adoptive transfer of γδ T cells isolated from 6 weeks-infected CD8^−/−^ JHT donors into Rag^−/−^ mice confers long-term protection against MCMV (135). Along the same line, γδ T cells isolated from 2 weeks-infected TCRα^−/−^ or wild-type C57BL/6 mice protected Rag^−/−^γc^−/−^ recipients against MCMV-induced death, whereas γδ T cells isolated from naïve mice failed to provide protection (41). Thus, effector and memory γδ T cells appear to be interesting candidate for adoptive cell transfer therapy against CMV.

In humans, Appay and colleagues interestingly analyzed γδ T cells in young adults (18–26 years old) who were thymectomized shortly after birth for cardiac surgery (122). This situation allows for the evaluation of the role of the post-birth thymus in the production of T cells responding to CMV. In contrast to control donors, no expansion of Vδ2^−^ γδ T cells could be observed in thymectomized patients. This setting is to our knowledge the only physiopathological situation in which HCMV is not associated with Vδ2^−^ γδ T cell expansion. This result strongly suggests that γδ T cells able to respond to HCMV are mainly produced in the thymus after birth. This is also consistent with the observation that HCMV infection in adults does not lead to the expansion of the public innate-like Vγ8Vδ1 TCR found in all HCMV-infected neonates (128).

Determining whether and which Ags are involved in γδ T cell expansion and activation requires further study and consideration. In contrast to long-term HCMV-induced Vδ2^−^ γδ T cells that display a restricted TCRδ repertoire (114, 119), the CDR3γ1 and γ4 length repertoire of murine γδ T cells was equivalent at 14 days in both infected and uninfected TCRα^−/−^ mice (41). Yet, some Vγ1 and Vγ2 T cell clones were enriched 28 days postinfection (135), while the TCRγ4 cells repertoire appeared oligoclonal even in naïve mice with no clear difference after infection (41, 135).

## Similarities Between γδ T and NK Cells in the Response to CMV

Despite shared features with αβ T cells, the function of γδ T cells responding to CMV cannot be considered as “merely” redundant to αβ T cells. In contrast to HCMV-specific αβ T cells, Vδ2^−^ γδ T cells express a panel of activating NK receptors, among which is the low affinity receptor for the constant fragment of IgG: CD16, which allows γδ T cells to recognize IgG-opsonized virus and induces the production of IFNγ without any prior TCR activation (Figures 1 and 2) (154). CD16 was also detected on MCMV-induced γδ T cells; moreover, MCMV and HCMV-induced γδ cells express the NKG2D (135); however, a role of this activating receptor in the recognition of HCMV-infected cells by Vδ2^−^ γδ T cells was ruled out (141). This is not surprising considering the evasion mechanisms developed by CMV to inhibit NKG2D ligand [MIC and UL16 binding protein (ULBP)] expression. γδ T cells that are selected *in vivo* by CMV and that undergo expansion, probably do not require NKG2D engagement to be stimulated. Conversely, neonate γδ T cells expanded during *in utero* CMV infection overexpress CD94 and NKG2C, which may be a response to the induction of HLA-E expression on HCMV-infected cells (127, 155). Vδ2^−^ γδ T cells from HCMV-infected transplant recipients or neonates also overexpress CD85j and diverse CD158 receptors when compared to both uninfected patients and HCMV-specific αβ T cells (Figure 2) (125, 139, 141). This high expression of inhibitory HLA-I receptors is probably important to regulate Vδ2^−^ γδ T cells prone to self-reactivity (see below), in a way similar to NK cell regulation. Consequently, the well-known evasion process developed by CMV consisting of MHC downregulation is probably an important trigger for the γδ T cell response to CMV infection. Another common feature between NK and Vδ2^−^ γδ T cells is the recognition of activating, stress-induced self-antigens (153). We have shown that HCMV-induced γδ T cells display a TCR-dependent dual reactivity against HCMV-infected cells and some tumor cells (141), which has been confirmed by another team (120). This dual reactivity relies on the recognition of stress-induced membrane self-antigens expressed on both HCMV-infected and cancer cells (142). This is reminiscent of the recognition of self-antigens on tumor cells or infected cells by NK cells, such as B7H6 recognized by NKp30 (156) or vimentin recognized by NKp46 (157).

## How Do γδ T Cells Control CMV Infection?

As documented below, CMV-induced γδ T cells are capable of (i) IFNγ and TNFα production that may synergize to inhibit CMV replication (158) and (ii) CMV-infected cell killing that may participate to CMV clearance (Figure 1).

In humans, Vδ2^−^ γδ T cell clones and lines isolated from peripheral blood of HCMV^+^ transplant recipients recognize HCMV-infected cells through their TCR. This leads to (i) the production of antiviral cytokines among which are TNFα and IFNγ, (ii) the killing of HCMV-infected cells, and (iii) the control of virus propagation *in vitro* (141). Similarly, Vγ8^+^Vδ1^+^ γδ T cell clones from HCMV-infected neonates exhibit IFNγ production *in vitro* when cultured with HCMV-infected cells (127). Vδ2^−^ γδ T cells can also use CD16 to recognize HCMV virions coated with anti-CMV antibodies and produce IFNγ that limit viral multiplication *in vitro* (154). Furthermore, HCMV-infected cells express caspase-1 inflammasomes and release IL-18. Engagement of the TCR on Vδ2^−^ γδ T cells controlled the direct innate immune sensing of IL-18, which enhanced cytotoxicity and IFNγ production by γδ T cells (159).

In mice, we performed *ex vivo* analysis (without prior stimulation) of IFNγ production and CD107a expression by γδ T cells isolated from organs during the course of MCMV infection in TCRα^−/−^ mice. The proportions of IFNγ^+^ and CD107a^+^ cells within γδ lymphocytes populations peaked at day 3 (IFNγ) and 7 (CD107a), then decreased until day 14 (41). Early production of IFNγ by γδ T cells was also evidenced in C57BL/6 mice (136) and is consistent with their capacity to rapidly sense and react against cellular dysregulation (108, 160). In αβ-T cell competent hosts, early production of IFNγ by γδ and NK cells may overrule the inhibitory function of viral proteins that interfere with MHC class I expression and enhance the antiviral efficiency of CD8^+^ T cells (161).

In TCRα^−/−^ infected mice, similar kinetics were observed when analyzing IFNγ-producing and cytotoxic NK cells (41). However, the latter largely outnumbered IFNγ-producing and cytotoxic effector γδ T cells, in accordance with the important role of NK cells in early MCMV control (32). Efficient control of viral load was observed upon γδ T cell transfer in recipient Rag^−/−^ mice treated with either anti-IFNγ or anti-IL-17 mAb (135). These results suggest that γδ T cell protective function does not involve IFNγ nor IL-17. However, further investigations are required to rule out their involvement in γδ T cell antiviral activity, because of the difficulty to completely inhibit cytokines with antibodies. Finally, Winkler’s group showed that γδ T cells isolated from 4 weeks-infected CD8^−/−^ JHT mice killed MCMV-infected mouse embryonic fibroblasts (MEFs), but not uninfected MEFs (135).

Altogether, these results suggest a biphasic response of γδ T lymphocytes during CMV infection: (i) an early phase mobilizing IFNγ-producing and cytotoxic γδ T cells, which act together with other immune effectors (particularly NK cells) for rapid and efficient viral control and (ii) the generation of EM γδ T cells able to protect the host in the long-term and whose effector and memory functions are under consideration in our laboratory.

## Clinical Interest of γδ T Cell Response to CMV

Different specificities make γδ T cells particularly tailored to respond to CMV. As mentioned above, the antigen specificity of γδ T cells is highly different from that of αβ T cells. We recently identified EPCR as a TCR ligand of a HCMV-expanded γδ T cell clone expressing a Vγ4Vδ5 TCR (142). Although EPCR presents some homologies with antigen-presenting molecules, i.e., MHC class I and CD1 molecules, its recognition is not dependent on lipid but rather relies on the direct binding of the TCR to EPCR itself. By contrast to MHC class I, HCMV infection of fibroblasts or endothelial cells does not affect EPCR expression (142). This suggests that γδ T cells are not impaired by the classical immune evasion processes developed by CMV to escape αβ T cells. It remains however to be tested whether host-virus co-evolution led to other γδ T cell-specific escape mechanisms. Localization of Vδ2^−^ γδ T cells in intestinal and lung epithelia and in the liver, i.e., sites of CMV entry and/or multiplication, is also probably important for their implication in the response to CMV.

Consequently, γδ T cells represent an interesting clinical target in the context of CMV infection. First, quantifying Vδ2^−^ γδ T cells in the blood is an easy assay to detect immune response to CMV in patients. Provided it is proven as reliable as CMV-specific αβ T cell detection, evidencing Vδ2^−^ γδ T cell expansion is more convenient and cheaper (one step direct staining in whole blood with only anti-CD3, anti-pan-delta, and anti-Vδ2 antibodies) than the detection of CMV-specific αβ T cells using MHC tetramers or activation by viral peptides. We recently revealed the prognostic value of their expansion to predict CMV-relapse in patient suffering from a first infection episode and treated by valganciclovir (162, 163). Second, their antiviral functions supported the development of new graft preparation procedures in stem cell transplantation. In recent clinical trials, depletion of whole T cells from the graft to avoid graft-versus-host disease was replaced by αβ T cell depletion (164). The goal of such procedure is to keep γδ T cells within the graft to prevent CMV infection and promote graft versus leukemia/lymphoma effect because of the γδ T cell reactivity against tumor cells (165), for review, see Ref. (166). Third, the ongoing identification of stress-induced self-antigens expressed by CMV-infected cells could pave the way toward the development of vaccination strategies using these antigens, in a similar way as what has been done in clinical trials using phosphoantigens activating Vγ9Vδ2 T cells (167). Fourth, development of cell therapy based on γδ T cells activated *in vitro* and reinjected in patients has been proposed in cancer (96, 168), but could also prove useful in CMV infection.

## Author Contributions

MC, CK and JD-M wrote the manuscript.

## Conflict of Interest Statement

The authors declare that the research was conducted in the absence of any commercial or financial relationships that could be construed as a potential conflict of interest.
